# Distinguishing Biotic vs. Abiotic Origins of ‘Bio’signatures: Clues from Messy Prebiotic Chemistry for Detection of Life in the Universe

**DOI:** 10.3390/life13030766

**Published:** 2023-03-13

**Authors:** Niraja V. Bapat, Sudha Rajamani

**Affiliations:** 1 Independent Researcher, Hyderabad 500046, Telangana, India; 2 Department of Biology, Indian Institute of Science Education and Research (IISER), Pashan, Pune 411008, Maharashtra, India

**Keywords:** origin of life, search for extraterrestrial life, prebiotic chemistry, abiosignatures, nonenzymatic reactions, astrobiology

## Abstract

It is not a stretch to say that the search for extraterrestrial life is possibly the biggest of the cosmic endeavors that humankind has embarked upon. With the continued discovery of several Earth-like exoplanets, the hope of detecting potential biosignatures is multiplying amongst researchers in the astrobiology community. However, to be able to discern these signatures as being truly of biological origin, we also need to consider their probable abiotic origin. The field of prebiotic chemistry, which is aimed at understanding enzyme-free chemical syntheses of biologically relevant molecules, could particularly aid in this regard. Specifically, certain peculiar characteristics of prebiotically pertinent messy chemical reactions, including diverse and racemic product yields and lower synthesis efficiencies, can be utilized in analyzing whether a perceived ‘signature of life’ could possibly have chemical origins. The knowledge gathered from understanding the transition from chemistry to biology during the origin of life could be used for creating a library of abiotically synthesized biologically relevant organic molecules. This can then be employed in designing, standardizing, and testing mission-specific instruments/analysis systems, while also enabling the effective targeting of exoplanets with potentially ‘ongoing’ molecular evolutionary processes for robust detection of life in future explorative endeavors.

## 1. Introduction

The search for life beyond Earth has intrigued humankind for a very long time. Nonetheless, we are still far from conclusively answering the questions related to whether we are alone in the Universe even after decades of enquiry. We only know one example of life as yet, and it is that which exists on Earth. However, with the discovery of a large number of exoplanets, the current count being at 5272 (https://exoplanets.nasa.gov/; accessed on 4 March 2023), the prospects of detecting extraterrestrial life look promising and more exciting than ever before.

An array of potential biosignatures have been tabulated for extraterrestrial life detection strategies [1]; most of these are drawn from our current knowledge of biology on Earth. Biosignatures are usually assigned based on a non-zero possibility of them being of potential biological origin. However, this does not necessarily preclude their chemical origin. Hence, it is important to consider that the detection of one or a few potential biosignatures might not necessarily confirm the possibility/presence of extraterrestrial life. For example, the abiotic production of oxygen might result in a false positive detection of life on candidate exoplanets [2]. In addition, abiotic organic matter may result in the formation of pseudomicrofossils and pseudomicrobialites, which can be mistaken as signatures reminiscent of existing or past biological activities [3]. In such cases, it is worthwhile to consider the abiotic origin/s of biologically important molecules in question. In the recently published report of the Committee on the Planetary Science and Astrobiology Decadal Survey of the National Academies of Sciences, Engineering, and Medicine, the importance of constructing a framework to interpret potential biosignatures, abiosignatures, false positives, and false negatives, as well as that of efforts to better understand those abiosignatures that may mimic the ones originating from biological sources, has been highlighted [4]. This report has marked the research pertaining to the differentiation between abiotic and biotic sources and processes in the context of biosignature detection, as an area of focus for the next decade. In addition, the European Astrobiology roadmap (AstRoMap) has also identified distinction between life and nonlife during biosignature detection, as a key objective that should be addressed in a more detailed and coordinated manner in the future [5].

Biologically important molecules can also arise from different abiotic reactions, thus, leading to an incorrect interpretation of the detected putative biosignatures. A plausible way to overcome this issue is to analyze the nuanced differences stemming from the biotic vs. chemical origin of these potential ‘bio’signatures. Simultaneously, it is also necessary to deduce/have some understanding of the geochemical settings on that particular planetary body, as the outcomes of organic reactions would vary based on various geochemical factors, including temperature, pH, reactant concentration, and availability of mineral ions.

To understand the likelihood of producing a given biologically pertinent molecule abiotically, which would result in ‘abiosignatures’ (i.e., signatures originating from abiotic processes), we need to gain insights from the events that led to the formation of first living cells (protocells) on the early Earth. The origin of life on Earth was an outcome of a complex set of non-trivial processes that allowed for the transition from the ‘nonliving’ chemistry realm to that of the ‘living’ biology realm. Since the first experimental demonstration of the synthesis of organic molecules relevant to biology in 1953 [6], researchers have been investigating this transition of complex organic chemistry to biology that eventually led to the formation of protocells. Research in this field of prebiotic chemistry has shed substantial light on the synthesis of many organic molecules relevant to biology, even in the absence of life. Such prebiotic chemical syntheses that are considered to have played an important role during the origin of life on Earth have some peculiar characteristics that are different from those associated with biosynthetic processes. Importantly, these prebiotic syntheses can provide clues regarding the extent to which complex organic molecules can be produced abiotically, thereby helping to rule out any abiotic sources when detecting potential biosignatures [7].

Herein, we discuss in detail the production and peculiar aspects of abiotically synthesized biologically important molecules, and emphasize the need for considering them while designing strategies to achieve convincing evidence of life’s presence on extraterrestrial cosmic bodies. This would also help in narrowing down those cosmic bodies where prebiotic chemistry might be currently active and may lead to the formation of living entities in the near or distant future. This would yield potential ‘targets’ for researchers to effectively direct their efforts of detecting extraterrestrial life. Importantly, the aim of this article is not to question the credibility of any of the actual or potential biosignatures, but to use our understanding of messy prebiotic chemistry to efficiently and productively maneuver our search for life beyond Earth.

## 2. Chemical Synthesis of Biologically Important Molecules

The main building blocks of life on Earth include monomers of nucleic acids, proteins, sugars, and lipids. Although all of these molecules are important for sustaining life, the two fundamental polymers required for the origin of life as we know it are nucleic acids, which carry information, and proteins, which perform the catalytic functions. Prebiotically relevant abiotic synthesis of amino acids has been known for almost 70 years. The seminal spark discharge experiment, which laid the foundation for research being undertaken in the area of prebiotic chemistry, demonstrated the successful formation of glycine from a mixture of water, methane, ammonia, and hydrogen, in addition to some other amino acids and many hydroxy acids [6]. Re-analysis of the original samples using modern techniques revealed the formation of many other amino acids during the original Urey–Miller synthesis experiment [8]. Furthermore, short chain peptides can be chemically obtained from these amino acids using mineral catalysis, condensing agents, or large amounts of salts [9]. A study has previously demonstrated the formation of up to 20-mer long peptides under alternate wet-and-dry conditions [10]. Moreover, studies have also demonstrated the formation of oligopeptides from diketopiperazines, which are usually obtained as the inhibitory byproducts of abiotic peptide synthesis [10,11]. Once formed, these peptides have also been shown to potentially pass on their information by acting as a template for self-replication [12].

As for nucleic acid monomers, their formation and subsequent polymerization have been achieved using chemical means. Small quantities of adenosine were obtained by direct heating of adenine with β-D-ribose [13]. In an alternative approach, synthesis of pyrimidine ribonucleotides was shown using pentose amino-oxazolines as an intermediate [14]. Once synthesized, the enzyme-free polymerization of such monomers and short oligomers has been shown to occur under varied conditions, including ice-water eutectic phases [15], clay-mineral assisted synthesis [16], alternate cycles of dehydration-rehydration [17], and supramolecular liquid crystalline assembly conditions [18]. Oligomerization of cyclic purine and pyrimidine nucleotides to yield short RNA oligomers has also been demonstrated under various conditions [19,20]. In addition, the abiotic synthesis of relatively long nucleic acids via RNA-catalyzed RNA elongation reactions has been successfully demonstrated [21,22]. The other two important types of biomolecules, viz. amphiphiles (e.g., fatty acids and lipids) and sugars, have been synthesized using chemical reactions such as Fischer–Tropsch Type (FTT) synthesis and formose reaction, respectively [23]. The starting reactants for both of these syntheses are small chain carbon-based molecules, which ultimately yield biologically important complex organic molecules such as fatty acids and pentose sugars.

The aforementioned examples highlight the fact that the mere detection of biologically important molecules would not necessarily confirm or even indicate the presence of active biology on an exoplanet or a planetary body or their satellites (e.g., Enceladus and Europa in our solar system). One has to factor in the possibility of chemical syntheses of such molecules, many of which have been demonstrated to occur under varied conditions, including at very high to even sub-zero level temperatures [24]. Additionally, the type and length of the abiotically produced biologically relevant molecules would also vary depending on the available reactants, chemical evolutionary stage, and geochemical settings on the extraterrestrial body under consideration. Hence, it is necessary to assess the success of abiotic reactions under diverse ‘messy’ chemical environmental settings. This is crucial for determining the extent to which potential biosignatures can, in fact, be produced in the absence of biology (i.e., prebiotically relevant abiotic signatures), which also could be an indication of an ongoing or past, chemical or molecular evolutionary processes.

## 3. Homochirality Is Atypical in Prebiotic Reactions

Many theories have been put forth in order to delineate the mechanism/s underlying the prominence of enantioselectivity in extant biology [25]. However, a comprehensive understanding of the same is still lacking. It is not clear whether the presence of homochiral compounds was an absolute necessity for the origin of life, or if it was an outcome of later events that occurred during the course of evolution. Given this, enantiopurity seems especially difficult to achieve using only chemical means. Nonetheless, the selective presence of either D- or L-enantiomers of certain biomolecules in cells is a very important hallmark of extant life on Earth. Efficient enzymatic machinery ensures almost exclusive incorporation of D-sugars and L-amino acids during most biochemical processes. Although this symmetry breaking implies under-utilization of monomers from the available chemical space, it also imparts selectivity to biological processes. Arguably, the simultaneous presence of enantiomers might have compromised the competence of biological enzymes in terms of substrate specificity, thereby resulting in their sub-optimal performance. In addition, the presence of racemic mixtures of monomers has also been shown to affect enzyme-free processes such as nonenzymatic template copying [26].

Prebiotically plausible syntheses of complex organic molecules usually yield both enantiomers. The formose reaction yields a mixture of both D- and L-isomers of different sugars, including ribose, threose, and erythrose [27]. Similar mixtures of sugars can be obtained by irradiating interstellar ice containing water, methanol, and ammonia [28]. Notably, the amino acids from the spark discharge experiments were obtained as racemic mixtures as well [8,29]. Additionally, frozen solutions of NH_4_CN kept at sub-zero temperatures for a prolonged period of time yielded both D- and L-isomers of amino acids [30]; such prolonged period of freezing is analogous to the conditions observed on Europa and other potential icy exoplanets. According to a conceptual model, racemization of the amino acids is expected in the hydrothermal production of organics on Enceladus [31]. Even extraterrestrial sources, such as carbonaceous meteorites, are known to contain only an enantiomeric excess of certain L-amino acids [32] and D-sugars [33]; the analyzed samples have never been observed to be entirely enantiopure.

Furthermore, both D- and L-isomers are known to participate in the abiotic reactions pertinent to biomolecules. For example, both D- and L-isomers of amino acids have been shown to get selectively adsorbed onto mineral surfaces [34]. Pertinently, their subsequent polymerization would lead to the formation of homochiral peptides of both handedness in the absence of any external selection pressure. Similarly, nucleotides containing either L-ribose or D-ribose can nonenzymatically polymerize in the absence or presence of a template [26]. This would potentially give rise to nucleic acid polymers with either chirality in the absence of selective pressure(s) exerted by biology. The presence of information-carrying molecules, specifically that of RNA, is considered to be of importance for the origin of life on Earth, especially due to its additional ability to facilitate catalytic reactions [35]. In this context, both right- and left-handed ribozymes have been shown to be capable of carrying out template copying [36], suggesting the presence and propagation of both the chiral forms of RNA on prebiotic Earth.

Thus, pertinent biologically important molecules obtained from abiotic reactions are not usually enantiopure. Observed enantiomeric excess may be an indication of how prebiotically relevant selection pressures might have shaped the evolutionary processes that had implications for life’s origins. This has been shown for protocellular systems, wherein relevant environmental pressures have been shown to affect the robustness and survival of certain molecular systems over the others [37,38]. Given these aforementioned aspects, our efforts should be aimed at detecting not just the biologically important complex organic molecules but also characterizing their chirality for discerning their abiotic vs. biotic origin [39]. The presence of messy racemic mixtures of such molecules might hint more at their abiological origin and need not necessarily be a resultant of past or present biological processes. Nonetheless, the presence of racemic mixtures may also represent ongoing chemical evolutionary processes, making the parent cosmic bodies that harbor such molecules interesting in terms of plausible detection of life or life-like entities in the future.

## 4. Prebiotic Syntheses Yield Heterogeneous Products

Enzyme-catalyzed reactions in living cells are usually optimized to yield a certain product or a set of products, from a predetermined set of substrates. These products usually have a defined chemical structure; for example, all nucleotides in DNA are linked to each other only by a 3′-5′ phosphodiester bond. The extent of product formation is further modulated according to the physiological needs of the cell. Thus, biochemical reactions are generally tailored to yield only the most useful set of products required by the cell at any given time. On the other hand, prebiotically relevant reactions tend to yield a plethora of products.

A case in point is the famous Urey–Miller synthesis that yields a mixture of isomers of different compounds. Using modern analytical techniques, it was found that the original samples from the spark discharge experiments contained a total of 22 different amino acids and 5 different amines [8]. More recent studies have shown that similar electric discharge experiments can also yield all four RNA nucleobases [40]. In addition, meteoritic samples have been shown to contain many nucleobase analogs and non-biological amino acids along with the nucleobases and amino acids that are observed in extant biology [41,42]. The formose synthesis does not yield only the ribose sugar; it instead yields a mixture of tetrose, pentose, hexose, and heptose sugars when starting from a simple carbon compound such as glycolaldehyde or glyceraldehyde [27]. The formose reaction also yields metabolically relevant carboxylic acids, including α-hydroxy acids, when carried out under alkaline conditions [43]. The FTT synthesis reactions, which are thought to be a plausible terrestrial source of fatty acids and lipids on early Earth, yield an array of compounds containing 2 to more than 30 carbon moieties. Usually, a complex mixture of alkanols, alkanoic acids, alkenes, and alkanes is obtained during FTT synthesis [44]. Even meteorites are now known to contain a highly complex mixture of amino acids, sugars and sugar-related compounds, nucleobases, carboxylic acids, and insoluble organic matter, thus hinting at the large chemodiversity present in Space [45].

It is conceivable that, when such diverse monomers get chemically linked to yield polymers, such as peptides and nucleic acids, these polymers would also be diverse in terms of their chemical bonds, sequence, structure, etc. In accordance with this, when co-oligomerization of different amino acids was attempted, varied sequences of peptides were obtained in the resultant product mix [10]. Similarly, a large number of sequence isomers were obtained during the co-oligomerization of amino acids and hydroxyl acids, resulting in the formation of depsipeptides [46]. When activated nucleic acid monomers undergo enzyme-free oligomerization, the resultant products typically contain both 3′-5′ and 2′-5′ phosphodiester bonds, the occurrence of which is not pre-determined [23]. Furthermore, the nonenzymatic replication of nucleic acids has been argued to result in numerous diverse sequences due to the intrinsic low fidelity of this process. On the other hand, prebiotic formation of non-biological nucleic acid monomers is possible using nucleobase analogs [47,48] and alternate sugars [49]. Such non-conventional monomers also seem to get linked to each other by a phosphodiester linkage [48]. Moreover, two chemically different monomers are also known to covalently interact with each other to yield new molecules capable of biologically relevant functions; the examples include peptide nucleic acids (PNAs), which can hybridize with other PNAs, RNAs, and/or DNAs via base pairing [50], and N-acyl amino acids capable of self-assembling to yield vesicles [51]. Given these studies, the existence of nucleic acids with alternate backbones that are capable of duplex formation and information transfer should also be factored in while looking for potential signatures of life. These studies clearly underline the possibility of the presence of a vast variety of peptide- and nucleic acid-like polymers in the absence of substantial biological constraints.

Biology seems to utilize only a limited chemical space when compared to the enormous variety of entities that could have been readily generated from un-intervened chemical syntheses [52]. It is very possible that, in biology, the most ‘useful’ molecules might have gotten selected very early on during the course of evolution, as is indicated by the presence of certain common set of biomolecules in all domains of known life. Retaining this set through billions of years would have also saved the efforts of re-inventing the fundamental structure of life, while still allowing for evolution. On the other hand, chemical systems do not necessarily show any preference for only a certain set of molecules, unless limited by reactivity or by environmental constraints. Furthermore, it is important to consider that extant biochemistry could be one of the many possible ways of sustaining life. Therefore, while assessing the molecules on or from extraterrestrial cosmic bodies (using, e.g., in situ or remote detection), it is worthwhile to account for the diversity of the chemical space and possibility of the presence of alternate polymers as well.

## 5. Nonenzymatic Reactions Are Less Efficient than Biotic Syntheses

The two main biopolymers fundamental to life on extant Earth, viz. nucleic acids and proteins, are of sufficiently long length that in turn supports their respective functions. In theory, there are four choices available for each position in a nucleic acid polymer and twenty choices available for each position in a peptide, i.e., 4^n^ and 20^n^ sequences possible for nucleic acids and proteins, respectively, for a polymer of length n. Therefore, the number of possible sequences would increase exponentially with the increasing length of these polymers. Increased sequence space would further mean higher chances of finding an optimal sequence for a suitable function. Additionally, a larger number of optimal sequences would help in adding complexity to living cells, thus facilitating their evolution. Concurrent with this is the fact that the functional genomes of multicellular organisms are observed to be much longer than those of prokaryotic cells [53]. Nevertheless, such long functional polymers can be formed and maintained inside living cells only due to the availability of a kinetically efficient enzymatic machinery.

On the contrary, nonenzymatic synthesis of peptides and nucleic acids usually yield short-chain products. This is mainly because of the intrinsic low rate of these condensation reactions as well as the presence of competing reactions such as hydrolysis and/or alternate product formation [54,55]. Higher product yields can be obtained by using catalysts such as clay surfaces [56], by chemically activating monomers to promote polymer formation [23], by concentrating monomers in really small spaces such as in the brine channels in ice-water eutectic phases [15], or by using combinations thereof. However, the length of the resultant polymer products is still not comparable to what is possible using biological enzymes. The length of the RNAs obtained using ribozyme-catalyzed reactions are also not close to that of the functional nucleic acid genomes observed in extant biology. Similar results are observed for FTT synthesis, wherein the yield of the amphiphiles decreases with the increase in the number of carbons [44]. In the formose reaction as well, sugars containing more than six carbons are usually obtained in miniscule quantities [27]. Thus, nonenzymatic chemical syntheses usually mainly yield a plethora of small molecular weight entities, and achieving complex longer-chain-length products using prebiotically relevant organic synthesis approaches has typically proven to be a daunting challenge.

In addition, nonenzymatic reactions follow equilibrium kinetics while extant biochemical reactions are usually observed in a disequilibrium state. Therefore, the distribution of chemical species would differ for products obtained using chemical synthesis vs. biological synthesis. In biology, the product from one reaction is usually used by other reactions as substrates, forming interdependent reaction networks. These tightly interacting networks ensure efficient formation and utilization of biologically important molecules, and might have been essential for the origin of life on Earth [57]. However, during chemical syntheses in a prebiotic pool, such strong interactions and feedback control, which represent an advanced stage of chemical evolution, might not be plausible unless the molecules are concentrated in a smaller volume that would allow for efficient molecular interactions. The well-orchestrated chemistry required to sustain life is, in general, more efficient and dynamic than the simple conversion of reactants to products. Hence, the detection of a simple conversion reaction under equilibrium state is likely to hint at a chemical evolutionary stage, even if the end product is a biologically relevant molecule.

## 6. Implications for the Search of Life in the Universe

The outcomes of prebiotic reactions studied thus far clearly highlight the possible abiotic origin/s of biologically relevant molecules. This is important to consider when assessing the source of a detected potential biosignature, which can in fact be a prebiotically relevant abiotic signature. If the concerned biologically relevant molecule/s is/are detected in high and enantiopure quantities in the absence of messy background molecules, such signature/s can indeed be considered as likely originating from a past or present biological activity. However, if these molecules are detected on an extraterrestrial body in the presence of a noisy background and in low quantities, such signature may likely have abiotic origin/s. In such cases, the presence of these complex organic molecules may not hint at the presence of life. Nonetheless, such signatures can indicate the presence of an active/ongoing chemical evolutionary process(es). Diverse prebiotically relevant chemical evolutionary paths are thought to have yielded the first living entities on the prebiotic Earth. Hence, the cosmic bodies where such potential biosignatures originating from abiotic sources (abiosignatures or complex chemical signatures) are detected may not just be interesting but will have direct implications for life’s eventual emergence on that particular extraterrestrial body, and hence for its detection in the future. The life detection community can thus focus on such cosmic bodies for planning and execution of targeted life detection endeavors in a concentrated and effective manner.

The accrued knowledge gathered from understanding chemical processes relevant to life’s origin on Earth and elsewhere can also be used to design and standardize mission-relevant instruments and related software for robotic/in situ as well as sample return missions. For example, as prebiotic syntheses usually yield a messy isomeric mixture of diverse molecules, it would be worthwhile to design instruments capable of analyzing the stereochemistry of a set of target molecules from the plethora of observable molecules. Understanding the yields of such prebiotic reactions will also help in setting the detection limits for various molecules on these analytical instruments. It is difficult to assess potential biosignatures without considering the planetary history, its geochemical environment, and the stage of chemical/biological evolution [58]. In this case, the products of prebiotic reactions that are carried out in the laboratory under various reaction conditions (mimicking the geochemical environment/s on cosmic bodies), and starting with different reactants (mimicking the reactant space heterogeneity and extent of chemical/biological evolution), may act as control samples for testing the mission instruments. In fact, the researchers engaged in understanding the origin of life need to come together and create a library of such molecules yielded from abiotic syntheses of biologically important molecules. These reactions are to be carried out in the laboratory and in natural analogue sites for delineating the various resultant species, which can then be used by the teams engaged in the detection of life in the Universe. Notably, eliminating the abiotic origin/s of a potential biosignature using such a library would still not necessarily confirm the presence of life because the signature may still have originated from a previously unknown chemistry and/or geochemical setting. Nonetheless, such a library will surely aid in designing more targeted efforts for detecting extraterrestrial life as well as understanding the limits of a planet’s habitability.

Certain cross-disciplinary research collaboration networks have recently been established to address important questions related to the emergence of life on Earth and beyond, and detection of life in the Universe. For example, the Prebiotic Chemistry and Early Earth Environments (PCE3) Consortium has been established to bring together early earth geoscientists and prebiotic chemists to better understand the processes involved in the emergence of life on Earth and other cosmic bodies. Researchers in the Network for life detection (NfoLD) are actively involved in undertaking research pertaining to life detection, including biosignature creation and preservation, as well as related technology development. The proposed library of abiotically synthesized biologically important molecules will benefit from the research carried out by scientists involved in collaborations such as the PCE3 consortium, while also being useful for scientists involved in collaborations such as NfoLD for designing effective life detection strategies. Importantly, we recommend that a collaborative data sharing platform be established between researchers from the origins of life and life detection communities (including biosignature assessment and instrument development), for facilitating concrete advancement in our understanding of life’s presence and distribution in the Universe. Such collaborations across diverse disciplines would greatly help in initiating a community-level dialogue that would positively support and refine our collective efforts in expanding life detection objectives.

## 7. Conclusions

With the continuous discovery of new exoplanets and advances in life detection technologies, the search for extraterrestrial life looks unprecedentedly promising. However, researchers should be prudent while detecting and segregating ‘complex chemical signatures’ or ‘abiosignatures’ from true ‘biosignatures.’ Experimental and theoretical studies carried out to decipher the origin of life on Earth could provide the requisite framework in this regard. Prebiotically relevant organic syntheses of biologically relevant molecules usually yield a heterogeneous spread of products containing many low molecular weight isomers. This contrasts with our understanding of how functional biomolecules are produced enzymatically in a living system. It can be expected that a minimal chaotic background would be detected for a ‘true’ biosignature as opposed to that observed for a prebiotically relevant abiosignature originating due to a divergent pool of chemicals yielded from messy prebiological reactions. To avoid this conundrum, a library of diverse molecules obtained from prebiotic reactions carried out under conditions reflecting various relevant geochemical settings, needs to be created in order to understand the extent to which biologically important complex organic molecules can be obtained in the absence of life. Such a library can be used to efficiently demarcate cosmic bodies with plausible active chemical evolution that is ongoing, while also allowing for designing and standardizing mission-specific instruments for a targeted and effective search for life beyond our pale blue dot.

## Data Availability

This article has no additional data.

## References

[1] Schwieterman E.W., Kiang N.Y., Parenteau M.N., Harman C.E., DasSarma S., Fisher T.M., Arney G.N., Hartnett H.E., Reinhard C.T., Olson S.L. (2018). Exoplanet biosignatures: A review of remotely detectable signs of life. Astrobiology.

[2] Harman C.E., Schwieterman E.W., Schottelkotte J.C., Kasting J.F. (2015). Abiotic O_2_ Levels on Planets around F, G, K, and M Stars: Possible False Positives for Life?. Astrophys. J..

[3] McMahon C., Cosmidis J. (2021). False biosignatures on Mars: Anticipating ambiguity. J. Geol. Soc..

[4] National Academies of Sciences, Engineering, and Medicine (2022). Origins, Worlds, and Life: A Decadal Strategy for Planetary Science and Astrobiology 2023–2032.

[5] Horneck G., Walter N., Westall F., Grenfell J.L., Martin W.F., Gomez F., Leuko S., Lee N., Onofri S., Tsiganis K. (2016). AstRoMap European Astrobiology Roadmap. Astrobiology.

[6] Miller S.L. (1953). A production of amino acids under possible primitive Earth conditions. Science.

[7] Hoehler T., Brinckerhoff W., Davila A., Marais D.D., Getty S., Glavin D., Pohorille A., Quinn R., Bebout L., Broddrick J. (2021). Groundwork for Life Detection. Bull. AAS.

[8] Johnson A.P., Cleaves H.J., Dworkin J.P., Glavin D.P., Lazcano A., Bada J.L. (2008). The Miller volcanic spark discharge experiment. Science.

[9] Rode B.M. (1999). Peptides and the origin of life. Peptides.

[10] Rodriguez-Garcia M., Surman A.J., Cooper G.J.T., Suárez-Marina I., Hosni Z., Lee M.P., Cronin L. (2015). Formation of oligopeptides in high yield under simple programmable conditions. Nat. Comm..

[11] Sasaki M., Miyagawa Y., Nonaka K., Miyanomae R., Quitain A.T., Kida T., Goto M., Honma T., Furusato T., Kawamura K. (2022). Nano-pulsed discharge plasma-induced abiotic oligopeptide formation from diketopiperazine. Sci. Nat..

[12] Lee D.H., Granja J.R., Martinez J.A., Severin K., Ghadiri M.R. (1996). A self-replicating peptide. Nature.

[13] Fuller W.D., Sanchez R.A., Orgel L.E. (1972). Studies in prebiotic synthesis: VI. Synthesis of purine nucleosides. J. Mol. Biol..

[14] Powner M.W., Gerland B., Sutherland J.D. (2009). Synthesis of activated pyrimidine ribonucleotides in prebiotically plausible conditions. Nature.

[15] Kanavarioti A., Monnard P.A., Deamer D. (2001). Eutectic phase in ice facilitates nonenzymatic nucleic acid synthesis. Astrobiology.

[16] Ferris J.P. (2006). Montmorillonite-catalysed formation of RNA oligomers: The possible role of catalysis in the origins of life. Philos. Trans. R. Soc. B.

[17] Rajamani S., Vlassov A., Benner S., Coombs A., Olasagasti F., Deamer D. (2007). Lipid-assisted synthesis of RNA-like polymers from mononucleotides. Orig. Life Evol. Biosph..

[18] Todisco M., Fraccia T.P., Smith G.P., Corno A., Bethge L., Klussmann S., Paraboschi E.M., Asselta R., Colombo D., Zanchetta G. (2018). Nonenzymatic Polymerization into Long Linear RNA Templated by Liquid Crystal Self-Assembly. ACS Nano.

[19] Dagar S., Sarkar S., Rajamani S. (2020). Geochemical influences on nonenzymatic oligomerization of prebiotically relevant cyclic nucleotides. RNA.

[20] Costanzo G., Saladino R., Botta G., Giorgi A., Scipioni A., Pino S., Di Mauro E. (2012). Generation of RNA molecules by a base-catalysed click-like reaction. ChemBioChem.

[21] Wochner A., Attwater J., Coulson A., Holliger P. (2011). Ribozyme-catalyzed transcription of an active ribozyme. Science.

[22] Attwater J., Wochner A., Holliger P. (2013). In-ice evolution of RNA polymerase ribozyme activity. Nat. Chem..

[23] Orgel L.E. (2004). Prebiotic chemistry and the origin of the RNA world. Crit. Rev. Biochem. Mol. Biol..

[24] Kitadai N., Maruyama S. (2018). Origins of building blocks of life: A review. Geosci. Front..

[25] Blackmond D.G. (2010). The origin of biological homochirality. Cold Spring Harb. Perspect. Biol..

[26] Joyce G.F., Visser G.M., Van Boeckel C.A., Van Boom J.H., Orgel L.E., Van Westrenen J. (1984). Chiral selection in poly(C)-directed synthesis of oligo(G). Nature.

[27] Zweckmair T., Böhmdorfer S., Bogolitsyna A., Rosenau T., Potthast A., Novalin S. (2014). Accurate analysis of Formose reaction products by LC–UV: An analytical challenge. J. Chromatogr. Sci..

[28] Meinert C., Myrgorodska I., De Marcellus P., Buhse T., Nahon L., Hoffmann S.V., Le Sergeant d’Hendecourt L., Meierhenrich U.J. (2016). Ribose and related sugars from ultraviolet irradiation of interstellar ice analogs. Science.

[29] Ring D., Wolman Y., Friedmann N., Miller S.L. (1972). Prebiotic synthesis of hydrophobic and protein amino acids. Proc. Natl. Acad. Sci. USA.

[30] Levy M., Miller S.L., Brinton K., Bada J.L. (2000). Prebiotic synthesis of adenine and amino acids under Europa-like conditions. Icarus.

[31] Steel E.L., Davila A., McKay C.P. (2017). Abiotic and biotic formation of amino acids in the Enceladus ocean. Astrobiology.

[32] Pizzarello S. (2006). The Chemistry of life’s origin:  A carbonaceous meteorite perspective. Acc. Chem. Res..

[33] Cooper G., Rios A.C. (2016). Enantiomer excesses of rare and common sugar derivatives in carbonaceous meteorites. Proc. Natl. Acad. Sci. USA.

[34] Hazen R.M., Filley T.R., Goodfriend G.A. (2001). Selective adsorption of L- and D-amino acids on calcite: Implications for biochemical homochirality. Proc. Natl. Acad. Sci. USA.

[35] Robertson M.P., Joyce G.F. (2010). The origins of the RNA world. Cold Spring Harb. Perspect. Biol..

[36] Sczepanski J.T., Joyce G.F. (2014). A cross-chiral RNA polymerase ribozyme. Nature.

[37] Sarkar S., Dagar S., Verma A., Rajamani S. (2020). Compositional heterogeneity confers selective advantage to model protocellular membranes during the origins of cellular life. Sci. Rep..

[38] Joshi M.P., Steller L., Van Kranendonk M.J., Rajamani S. (2021). Influence of Metal Ions on Model Protoamphiphilic Vesicular Systems: Insights from Laboratory and Analogue Studies. Life.

[39] Glavin D.P., Burton A.S., Elsila J.E., Aponte J.C., Dworkin J.P. (2020). The Search for Chiral Asymmetry as a Potential Biosignature in our Solar System. Chem. Rev..

[40] Ferus M., Pietrucci F., Saitta A.M., Knížek A., Kubelík P., Ivanek O., Shestivska V., Civiš S. (2017). Formation of nucleobases in a Miller–Urey reducing atmosphere. Proc. Natl. Acad. Sci. USA.

[41] Callahan M.P., Smith K.E., Cleaves H.J., Ruzicka J., Stern J.C., Glavin D.P., House C.H., Dworkin J.P. (2011). Carbonaceous meteorites contain a wide range of extraterrestrial nucleobases. Proc. Natl. Acad. Sci. USA.

[42] Elsila J.E., Aponte J.C., Blackmond D.G., Burton A.S., Dworkin J.P., Glavin D.P. (2016). Meteoritic amino acids: Diversity in compositions reflects parent body histories. ACS Cen. Sci..

[43] Omran A., Menor-Salvan C., Springsteen G., Pasek M. (2020). The Messy Alkaline Formose Reaction and Its Link to Metabolism. Life.

[44] McCollom T.M., Ritter G., Simoneit B.R.T. (1999). Lipid synthesis under hydrothermal conditions by Fischer- Tropsch-Type reactions. Orig. Life Evol. Biosph..

[45] Ruf A., D’Hendecourt L.L.S., Schmitt-Kopplin P. (2018). Data-Driven Astrochemistry: One Step Further within the Origin of Life Puzzle. Life.

[46] Forsythe J.G., Petrov A.S., Millar W.C., Yu S.-S., Krishnamurthy R., Grover M.A., Hud N.V., Fernández F.M. (2017). Surveying the sequence diversity of model prebiotic peptides by mass spectrometry. Proc. Natl. Acad. Sci. USA.

[47] Chen M.C., Cafferty B.J., Mamajanov I., Gállego I., Khanam J., Krishnamurthy R., Hud N.V. (2014). Spontaneous prebiotic formation of a β-Ribofuranoside that self-assembles with a complementary heterocycle. J. Am. Chem. Soc..

[48] Mungi C.V., Singh S.K., Chugh J., Rajamani S. (2016). Synthesis of barbituric acid containing nucleotides and their implications for the origin of primitive informational polymers. Phys. Chem. Chem. Phys..

[49] Fialho D.M., Clarke K.C., Moore M.K., Schuster G.B., Krishnamurthy R., Hud N.V. (2018). Glycosylation of a model proto-RNA nucleobase with non-ribose sugars: Implications for the prebiotic synthesis of nucleosides. Org. Biomol. Chem..

[50] Frenkel-Pinter M., Samanta M., Ashkenasy G., Leman L.J. (2020). Prebiotic peptides: Molecular hubs in the origin of life. Chem. Rev..

[51] Joshi M.P., Sawant A.A., Rajamani S. (2021). Spontaneous emergence of membrane-forming protoamphiphiles from a lipid-amino acid mixture under wet-dry cycles. Chem. Sci..

[52] Meringer M., Cleaves H.J. (2017). Exploring astrobiology using in silico molecular structure generation. Phil. Trans. R. Soc. A.

[53] Lynch M., Conery J.S. (2003). The origins of genome complexity. Science.

[54] Bapat N.V., Rajamani S. (2018). Templated replication (or lack thereof) under prebiotically pertinent conditions. Sci. Rep..

[55] Mungi C.V., Bapat N.V., Hongo Y., Rajamani S. (2019). Formation of abasic oligomers in nonenzymatic polymerization of canonical nucleotides. Life.

[56] Huang W., Ferris J.P. (2006). One-step, regioselective synthesis of up to 50-mers of RNA oligomers by Montmorillonite catalysis. J. Am. Chem. Soc..

[57] Cronin L., Walker S.I. (2016). Beyond prebiotic chemistry. Science.

[58] Barge L.M., Rodriguez L.E., Weber J.M., Theiling B.P. (2022). “Determining the Biosignature Threshold” for Life Detection on Biotic, Abiotic, or Prebiotic Worlds. Astrobiology.

